# Development and use of health outcome descriptors: a guideline development case study

**DOI:** 10.1186/s12955-020-01338-8

**Published:** 2020-06-05

**Authors:** Tejan Baldeh, Zuleika Saz-Parkinson, Paola Muti, Nancy Santesso, Gian Paolo Morgano, Wojtek Wiercioch, Robby Nieuwlaat, Axel Gräwingholt, Mireille Broeders, Stephen Duffy, Solveig Hofvind, Lennarth Nystrom, Lydia Ioannidou-Mouzaka, Sue Warman, Helen McGarrigle, Susan Knox, Patricia Fitzpatrick, Paolo Giorgi Rossi, Cecily Quinn, Bettina Borisch, Annette Lebeau, Chris de Wolf, Miranda Langendam, Thomas Piggott, Livia Giordano, Cary van Landsveld-Verhoeven, Jacques Bernier, Peter Rabe, Holger J. Schünemann

**Affiliations:** 1 Department of Health Research Methodology, Evidence and Impact, Michael G. DeGroote Cochrane Canada and GRADE Centres, McMaster University, 1280 Main Street West, Hamilton, ON L8N 4K1 Canada; 2 Michael G. DeGroote Cochrane Canada and MacGRADE Centres, 1280 Main Street West, Hamilton, Ontario L8S 4K1 Canada; 3 European Commission, Joint Research Centre (JRC), Via E. Fermi 2749 – TP 127, I-21027 Ispra, VA Italy; 4 Department of Oncology, McMaster University, Hamilton, Canada; 5 Cochrane GRADEing Methods Group, 1280 Main Street West, Hamilton, Ontario L8S 4K1 Canada; 6 European Commission Initiative on Breast Cancer Guidelines Development Group, European Commission, JRC, Ispra, Italy; 7 Private Group Practice for Radiology, Radiologie am Theater, Paderborn, Germany; 8 Department for Health Evidence, Radboud University Medical Center, PO Box 9101, 6525 EZ HB Nijmegen, The Netherlands; 9 Dutch Expert Centre for Screening, PO Box 6873, 6503 GJ Nijmegen, the Netherlands; 10 Centre for Cancer Prevention, Queen Mary University of London, Charterhouse Square, London, EC1M 6BQ United Kingdom; 11 Cancer Registry of Norway, PO 5313, Majorstua, 0304 Oslo, Norway; 12 Oslo Metropolitan University, Pilestredet 48, 0167 Oslo, Norway; 13 Umeå University, 90187 Umeå, Sweden; 14 Leto Gynecological-Surgical and Obstetrical Clinic, 18, Avenue Kifissias, 11526 Athens, Greece; 15 Cardiff and Vale Breast Centre, University Hospital Llandough, Llandough, United Kingdom; 16 EUROPA DONNA - The European Breast Cancer Coalition, Piazza Amendola 3, 20149 Milan, Italy; 17 National Screening Service, Kings Inns House, 200 Parnell Street, Dublin, D01 A3Y8 Ireland; 18 Epidemiology Unit, Azienda Unità Sanitaria Locale – IRCCS di Reggio Emilia, Via Amendola 2, 42122 Reggio Emilia, Italy; 19 School of Medicine, University College Dublin, BreastCheck, Irish National Breast Screening Programme, St. Vincent’s University Hospital, Elm Park, Dublin 4, Ireland; 20 Insitute of Global Health, University of Geneva, chemin des Mines 9, 1202 Geneva, Switzerland; 21 Department of Pathology, University Medical Center Hamburg-Eppendorf, Hamburg, Germany; 22 University Medical Center Hamburg-Eppendorf, Martinistr. 52, 20246 Hamburg, Germany; 23 Department of Clinical Epidemiology, Biostatistics and Bioinformatics, Amsterdam UMC, University of Amsterdam, Meibergdreef 9, Amsterdam, The Netherlands; 24 CPO Piedmont-AOU Citta della Salute e della Scienza, via Cavour 31, 10131 Turin, Italy; 25 Department of Medicine, McMaster University, 1280 Main Street West, Hamilton, Ontario L8S 4K1 Canada

**Keywords:** Health outcomes, Health states, Health utility, Guideline methodology

## Abstract

**Background:**

During healthcare guideline development, panel members often have implicit, different definitions of health outcomes that can lead to misunderstandings about how important these outcomes are and how to balance benefits and harms. McMaster GRADE Centre researchers developed ‘health outcome descriptors’ for standardizing descriptions of health outcomes and overcoming these problems to support the European Commission Initiative on Breast Cancer (ECIBC) Guideline Development Group (GDG). We aimed to determine which aspects of the development, content, and use of health outcome descriptors were valuable to guideline developers.

**Methods:**

We developed 24 health outcome descriptors related to breast cancer screening and diagnosis for the European Commission Breast Guideline Development Group (GDG). Eighteen GDG members provided feedback in written format or in interviews. We then evaluated the process and conducted two health utility rating surveys.

**Results:**

Feedback from GDG members revealed that health outcome descriptors are probably useful for developing recommendations and improving transparency of guideline methods. Time commitment, methodology training, and need for multidisciplinary expertise throughout development were considered important determinants of the process. Comparison of the two health utility surveys showed a decrease in standard deviation in the second survey across 21 (88%) of the outcomes.

**Conclusions:**

Health outcome descriptors are feasible and should be developed prior to the outcome prioritization step in the guideline development process. Guideline developers should involve a subgroup of multidisciplinary experts in all stages of development and ensure all guideline panel members are trained in guideline methodology that includes understanding the importance of defining and understanding the outcomes of interest.

## Introduction

Healthcare guidelines aim to support healthcare professionals, recipients of care and policy makers in making best decisions for care. Guidelines, and the research evidence that supports them, are not without risk of bias [1–3]. If bias is not managed appropriately it is possible that guideline developers could formulate an inappropriate recommendation, or guideline end-users could misinterpret a recommendation. One of the ways which the guideline development community has tried to manage bias is by recommending that the certainty of the evidence be rated and presented in the guideline [1, 3–7]. The goal of the exercise is to identify bias and improve the transparency of developers’ considerations that are used to formulate a recommendation. The implications of doing this are that guideline end-users may decide for themselves how and when to apply guidelines for their own needs.

The Grading of Recommendations, Assessment, Development, and Evaluation (GRADE) approach is a framework that is widely used by guideline developers and other organizations to systematically evaluate the quality of evidence, determine the strength of healthcare recommendations, and improve transparency of guideline development methods [8]. One of the aims of the GRADE approach is to minimize the complexity while increasing transparency of evidence evaluation. In part, GRADE does this by guiding developers to consider only the health outcomes which matter to patients most. The rating and selection of important health outcomes occurs before the search for evidence because it helps narrow the search. Collectively, guideline groups generate a list of relevant health outcomes. Guideline developers using GRADE individually rate each outcome according to how important they think it might be to patients in the given healthcare scenario [9]. Outcomes are rated on a 1 to 9 scale (1–3 = low importance for decision making, 4–6 = important, but not critical for decision making, 7–9 = critical for decision making) [10]. GRADE dictates that the outcomes with the highest average rating (rated at least “important”) should be chosen for consideration of that healthcare question. These outcomes, and the corresponding evidence, are presented to guideline panels in GRADE evidence tables that summarize the key information of a systematic review and support decision-making [11–14]. The importance rating exercise intends to mitigate several challenges in guideline development. It orients panel members to the task of focusing on outcomes that matter to patients, thus reducing the number of outcomes deemed to be patient-important, identifies the level of agreement for the outcome of interest, and indicates the relative importance of the beneficial and harmful outcomes (e.g. within the “critically important” category an outcome rated as 9 will be more important than an outcome rated as 7).

Health utility ratings are used similarly in a guideline panel’s harm-benefit analysis of health outcomes [15]. Health utility is a measure of patients’ values attached to the outcomes [16]. Outcome-specific health utility ratings are often not available or are not applicable to certain target populations [17]. Therefore, panels sometimes rate the health utility of outcomes internally to most accurately measure their collective views on the relative benefits and harms of each outcome. For instance, guideline panel members may rate the outcome on the validated Visual Analogue Scale (VAS) which is anchored by the health states “dead” and “full health” at 0 and 100 respectively.

However, we identified a fundamental problem with consideration of outcomes and calibration of the importance and utility rating scales. That is, panel members often have implicit different definitions of health outcomes that can lead to differences in importance ratings, utility ratings, and final panel recommendations. In fact, a recent observation in the Guidelines Development Group (GDG) that is developing the European guidelines for breast cancer screening and diagnosis within the European Commission Initiative on Breast Cancer (ECIBC) was the impetus for this study. When asked to define health outcome “over-diagnosis of breast cancer” in a concealed fashion, each GDG member provided a considerably different description of the outcome. However, clear definitions and agreement by a guideline panel on what constitutes an outcome is required to search for evidence, balance benefits and harms, communicate with the public, and conduct research. Furthermore, to promote transparency of guideline development methods, guideline end-users require clear explanations of what constitutes each important outcome.

To tackle the issue of standardizing definitions of health outcomes in the ECIBC guideline recommendations (ECIBC guidelines), we utilized a novel template for ‘health outcome descriptors’ developed by researchers at McMaster GRADE Centre. The template is based on the concept of ‘health states’ or ‘clinical marker states’ [18, 19]. Our health outcome descriptors are primarily intended to support the generation of recommendations by guideline developers and promote understanding of development methods by guideline end-users secondarily. Here, we describe the development and use of these health outcome descriptors in the context of the ECIBC guidelines. The purpose of this case study was to determine which aspects of the development, content and use of health outcome descriptors are valuable to guideline developers broadly. We describe lessons learned to improve the structure of the tool and provide guidance for the future development and use of health outcome descriptors.

## Methods

### General methods

**Fig. 1:**
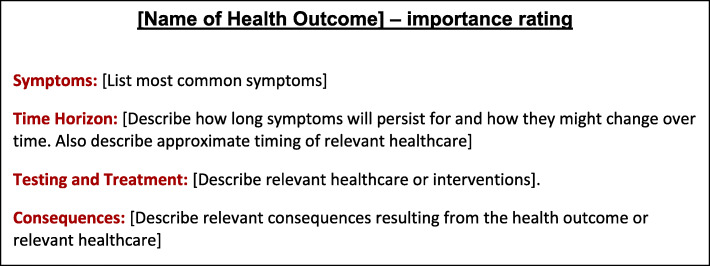
Draft Template for Development of Health Outcome Descriptors

We conducted a case study of the development of health outcome descriptors in the context of the European guidelines for breast cancer screening and diagnosis. We selected a case study design to systematically collect quality feedback from guideline developers involved in the process of health outcome descriptor development. The development of the health outcome descriptors was based upon proposed guidelines for their development [20]. We developed first drafts of the health outcome descriptors for the ECIBC guidelines using a template (Fig. 1). Throughout development, GDG members provided feedback on the drafts and development process. This was done through three rounds of semi-structured interviews and online written feedback. Iterative changes were made to the content and format of the health outcome descriptors based upon the observations of McMaster University researchers and extensive GDG feedback. In between rounds of feedback, GDG members also completed two online health utility assessments. We analyzed the utility scores to quantitively assess whether the development process had an impact on harmonization of outcome definitions as well as values and preferences the GDG had towards the health outcomes.

### Participants

We formed a steering committee to coordinate the development of the health outcome descriptors for the European guidelines for breast cancer screening and diagnosis consisting of five researchers: four health methods researchers (HS, NS, PM, ZSP) and one graduate student with training in health sciences (TB).

An opportunity sample of the guidelines development group (GDG) volunteered to participate in the development of the health outcome descriptors in varying capacities. Ten of the thirty GDG members took part in 14 semi-structured interviews to collect feedback on the development methods, content, use, and implementation plans for ECIBC health outcome descriptors. Of those interviewed more than once, one panel member was interviewed three times and two were interviewed twice. Separately, twelve of the thirty GDG members participated in each of the online utility rating surveys (which were sent to every panel member), respectively. Six of those GDG members participated in both surveys.

GDG members were clinicians, epidemiologists, cancer scientists, methodologists, economists, and patients. All GDG members, including those participating in this study, were selected for the panel via an open call by DG Sante to develop the ECIBC guidelines [21]. Each GDG member declared their interests to the ECIBC as part of their agreement to participate in the guideline development. Every GDG member was requested to participate all aspects of health outcome descriptor development for this study. However, participation in this study was voluntary. Signed consent was obtained from all those providing feedback and this study was approved by the Hamilton Integrated Research Ethics Board (HiREB).

### Template of health outcome descriptors

We utilized a draft template (Fig. 1) for health outcome descriptors [18–20]. The format was purposefully designed to be concise; written at a Grade 8 reading level (as indicated by the Flesch–Kincaid readability tests) from the perspective of the healthcare recipient, who is the primary beneficiary of any healthcare guideline. The template included 4 bulleted domains: “Symptoms”, “Time Horizon”, “Treatment and Testing”, and “Consequences”.

### Development of draft health outcome descriptors

**Fig. 2:**
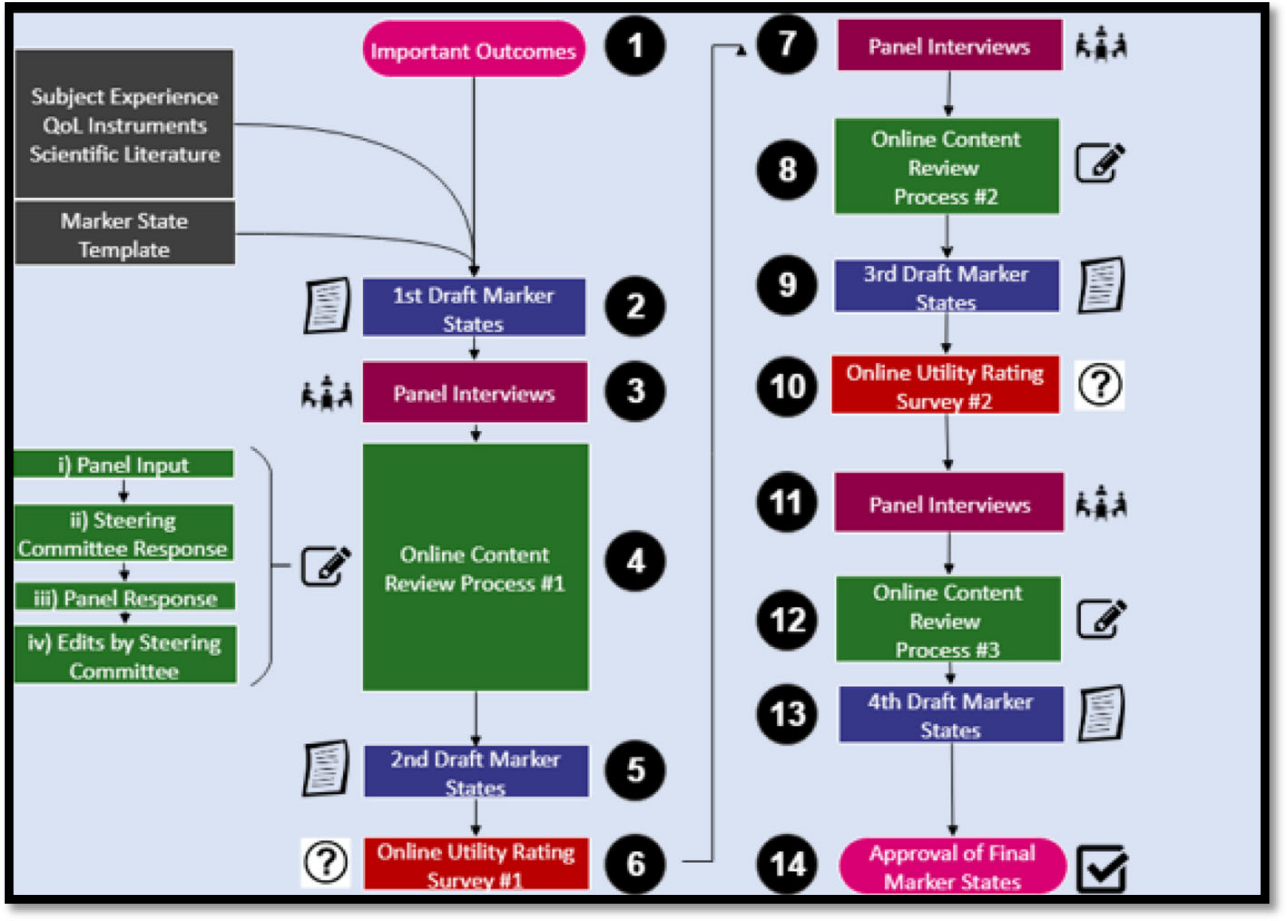
Health outcome descriptor development process. McMaster researchers developed first drafts of the health outcome descriptors using a template and relevant source material which were reviewed by the entire steering committee. Nineteen volunteers from the GDG panel provided feedback on the drafts in semi-structured interviews and/or online review. This was done through three rounds of semi-structured interviews and online written feedback. Iterative changes were made to the content and format of the health outcome descriptors based upon the observations of the steering committee and GDG feedback collected. In between rounds of feedback, a subset of GDG members also completed two online health utility assessments

The methods for development of the first draft health outcome descriptors are summarized in Fig. 2 (steps 1–3). Realizing the need to harmonize understanding of the ECIBC health outcomes, the steering committee used the draft template (Fig. 1) to write 24 draft health outcome descriptors relevant to the healthcare questions for the ECIBC guidelines. The outcomes chosen for health outcome descriptor development in this study were selected because they had already been prioritized for the ECIBC guidelines before the start of this study. If health outcome descriptors are used in practice it should typically precede rating for importance, to ensure harmonization, accuracy and transparency of the rating exercise. To populate the draft template, the steering committee utilized information from quality of life instruments, scientific literature, and collective subject experience [22–31].

### Refinement of health outcome descriptor content and structure

Figure 2 summarizes our methods for reviewing the development of the health outcome descriptors (steps 4–10). After the steering committee completed internal development of the drafts, ECIBC GDG members were invited to provide feedback on the development methods, content, and structure of the health outcome descriptors by means of semi-structured interviews and online comments. All 30 GDG members were asked, and 19 participated in some capacity. Ten volunteered to participate in individual semi-structured interviews at the JRC-Ispra location and the subsequent online refinement. Separately, nine of 30 GDG members volunteered written comments only. All interviews were conducted at quarterly GDG meetings, by the same interviewer (TB), using the same list of prompting questions with transcription for analyses. Whenever possible, we repeated interviews with available panel members at different meetings to get their feedback throughout development. During the written online refinement, GDG members could actively discuss content issues with other ECIBC GDG members. We developed second drafts of all health outcome descriptors after reviewing the GDG’s feedback and making the relevant changes to the health outcome descriptors when there were factual errors or important omissions in content. When unsure whether to make changes based upon GDG feedback, the steering committee looked for supporting literature before approving the changes. We then held two additional rounds of GDG feedback (each having an interview and online component) and made edits using the same approach to develop a third and fourth draft, respectively. Throughout the development process, we ensured that all health outcome descriptors were reviewed by at least one member of the GDG. After each round of feedback with the GDG volunteers, the drafts were presented to the entire GDG for review or approval. After each presentation, the GDG discussed specific feedback and concerns about the health outcome descriptor development process with the steering committee.

### Online utility rating surveys

In parallel to health outcome descriptor development, the steering committee conducted online surveys to elicit health utilities from the GDG for the 24 health outcomes using a VAS. The purpose of this exercise was to validate health outcome descriptors for evaluating the health utility of health outcomes. On the 0 to 100 VAS, 0 was anchored at “dead” and 100 at “full health” [18, 19]. The steering committee administered two surveys to the entire GDG immediately after development of the second and third health outcome descriptor drafts, respectively. Each survey was to be completed individually. Thus, by design, the GDG members that participated rated the health utility of each health outcome twice (once per survey). The most current versions of the health outcome descriptors were used to describe all health outcomes in the surveys, including the VAS anchors. The steering committee made iterative changes to the survey instructions based upon thematic analysis of the GDG’s interview feedback.

### Data analysis

We conducted thematic analysis of the transcribed GDG interviews and utility surveys in six steps [32] using NVIVO version 11 software. First, two McMaster GRADE Centre researchers (TB, GPM) reviewed the interview transcripts and survey feedback. Second, each reviewer independently coded the material. Third, coding was reviewed to identify common themes. Care was taken to note the respective timing of the themes in development, and how they changed over time. Fourth, the two reviewers met to pool the themes and ensure that the codes were appropriate for each theme, and then they discussed and agreed on the refinement of the themes. Finally, the first author applied the themes during manuscript drafting for review by the steering committee.

We conducted all quantitative analyses of the health utility ratings using IBM SPSS version 20. For the descriptive analysis, we calculated the outcome-specific mean utility ratings per survey, and corresponding standard deviation for each health outcome descriptor. If our health outcome descriptors were effective for harmonizing the understanding of outcomes, we expected to observe a reduction in variance of mean health utility scores across outcomes. For each outcome we performed Levene’s F-tests to assess whether the variance in mean utility ratings for both surveys were equivalent to one another. The rates and outcomes were the same for both surveys, so we hypothesized that there would be less variance over time if, through the iterative process, the content of the health outcome descriptors had improved. We expected to observe an improvement in inter-rater agreement in the second utility rating survey because the changes in the health outcome descriptors would better represent the values of the panel. To assess the agreement in utility scores between raters on the VAS, we calculated the intraclass correlation coefficient (ICC) for raters in each survey using a two-way random effects model.

## Results

### Health outcome descriptors

**Fig. 3:**
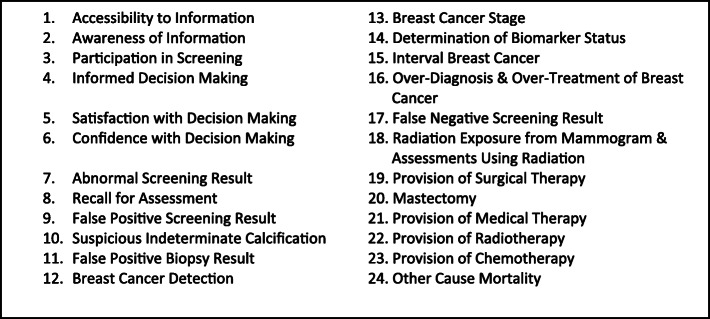
List of Health outcome descriptors developed for ECIBC

**Fig. 4:**
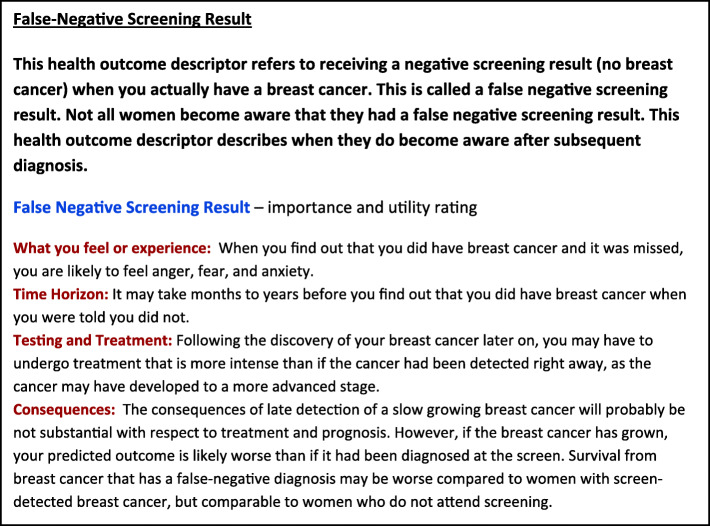
Example Health outcome descriptor developed for ECIBC

We developed 24 health outcome descriptors (Fig. 3); each was approved by the ECIBC’s GDG. An example health outcome descriptor is provided in Fig. 4 and the full ECIBC guideline health outcome descriptors are presented both in the Appendix and the GRADEpro Guideline Development Tool health outcome descriptor database (ms.gradepro.org). This database already houses health outcome descriptors for nearly 100 outcomes for several conditions and developers are invited to submit their work to enhance the database [33].

### ECIBC GDG interview feedback

Six, four, and four interviews were conducted after the development of the first, second, and third health outcome descriptor drafts, respectively. The thematic analysis of the semi-structured interview transcripts revealed six themes.

#### Theme 1: health outcome descriptor development process

Overall, most GDG members felt that the methods used to develop the health outcome descriptors in this study were appropriate. Specifically, most interviewees described the refinement process as acceptable, quick, and effective for improving the quality of the content to an acceptable level.

Despite repeated presentations at GDG meetings, participants felt that the purpose of health outcome descriptor development in the context of this study was not made clear to them. Therefore, GDG members described insufficient training on the development process and aims of health outcome descriptors as initial barriers to participating in their development.

#### Theme 2: comprehensibility of health outcome descriptors

Most members of the GDG felt that the wording of the health outcome descriptors became relatively clear and consistent by the end of the refinement process. Reading level and emotional sensitivity emerged as important factors for facilitating the use of health outcome descriptors by guideline end-users. Some GDG members felt that the reading level should be relatively high because end-users might feel intellectually insulted by a low reading level:

*“The reading level should be increased. We cannot offend women.”*

Other members suggested that the content should be at a lower reading level to facilitate use of health outcome descriptors by less educated members of the public:

*“If [health outcome descriptors] are to be used by the broad public I think they need re-wording for someone of a lower literacy level.”*

The panel was split regarding whether direct language and mention of negative health effects should be avoided to improve emotional sensitivity of the health outcome descriptors. There was mixed feedback about whether multiple versions of health outcome descriptors (e.g. for healthcare recipients, panel members, healthcare professionals, etc.) should be developed for a single guideline based upon the appropriateness of wording and emotional sensitivity for specific end-user populations.

#### Theme 3: data presentation

Throughout development, it was the opinion of the steering committee that the GDG members shied away from including information that might make the health outcome descriptors too specific (e.g. stating precise wait times, uncommon side effects of medical procedure, etc.). Some GDG members became concerned that the information in the health outcome descriptors would only be relevant to a small population of those experiencing a health outcome if the information was too specific. The use of descriptive statistics emerged as an important factor in improving the generalizability of health outcome descriptors. GDG members felt that use of the averages for representing quantitative information in the health outcome descriptors did not represent the variety of possibilities that an individual could experience for any health outcome:

*“Whether it be weeks, days or months; there can be a lot of variation [in timing of symptoms]. So, it seems a bit artificial to state a specific time”*

The health outcome descriptors were described as more representative when quantitative information was presented with only the minimum and maximum feasible data values, typically in the form of time periods and ranges.

#### Theme 4: health outcome descriptor structure & content

Overall, GDG members deemed the format of health outcome descriptors to be acceptable. All participants thought that the domains were comprehensive, presented in a logical order, and easily identifiable. However, they explained that the descriptor of the “Symptoms” domain should be changed to make it more intuitive.

Several GDG members acknowledged that the “Consequences” domain was necessary for describing any outcome. However, some felt that there was little variation across all the outcomes. However, it is likely that outcomes for a specific problem or disease and narrow interventions will incur similar consequences.

One GDG member mentioned that the “Testing and Treatment” domain was not appropriate for outcomes for screening programs and preventive efforts because healthcare recipients might not receive treatment:

*“Most women that go for screening will not enter any kind of diagnostic efforts, let alone be treated. So, I find it very artificial to be reading up on health outcome descriptors that are directly related to the screening process, and then being pushed [to consider] the treatment area”*

That GDG member recommended separating “Testing” and “Treatment” into two domains and explicitly stating when the domains are not relevant.

#### Theme 5: using health outcome descriptors

During early development, the aims and final users of the health outcome descriptors were not clearly understood by GDG members. However, as some GDG members became more familiar with health outcome descriptors they agreed that they could be useful for consolidating understanding of outcomes among guideline developers, facilitating panel discussion, and improving the transparency of guideline methods. One GDG member reflected upon the development process as follows:

*“I think [health outcome descriptors] have been very valuable to the [GDG] because it has made us discuss with you, and the rest of the [GDG], what we really mean.”*

There was agreement among GDG members that if health outcome descriptors are used during panel discussion, panel chairs should refer to outcome definitions. Some of the GDG felt that if health outcome descriptors were to be used externally, attaching them to the recommendations or publishing them online was important for making them available to end-users.

#### Theme 6: utility rating survey

Most GDG members indicated that the first online survey was problematic and difficult to complete. Much of the difficulty they described referenced the inappropriateness of the VAS anchors (“dead” and “full health”) for rating the health utility of outcomes which had emotional and psychological implications as opposed to physical (e.g. the health outcome descriptor ‘Awareness to Information’):

*“The survey was problematic for me. I tried to complete it honestly but some of the [outcomes], did not lend themselves to the scale of dead and full health.”*

After the first survey, it emerged that some participants were inappropriately making attribute-based comparisons (e.g. considering only physical or mental or emotional symptoms) or comparing the total number of implications described in each health outcome descriptor. The fact that a holistic strategy should be used to rate how the physical, emotional, and mental implications might affect overall health relative to the anchors was not sufficiently clear to participants Therefore, the instructions in the second survey were modified to better direct GDG members through the health utility rating process. Other comments from GDG members suggested that difficulties with the VAS may have manifested from problems with the initial outcome prioritization exercise carried out by the GDG:

*“Some of [the outcomes] … why on earth are there health outcome descriptors for that? It becomes hard to rate if you don’t see [the outcome] as important”*

### Utility rating survey scores

2The mean utility ratings for each survey, the results of the pairwise comparison, and variability comparison are presented in Table 1. We attempted to evaluate if the health outcome descriptor revisions had important impact on the health utility ratings. Between the first and second surveys, we observed an increase in the mean scores of 14 outcomes and a decrease in 10 outcomes when results from all participants were analyzed. The variability, that is the magnitude of the standard deviation, of the ratings improved in 21 pairs and it remained similar in 2 pairs. In one health outcome descriptor the standard deviation increased slightly. The ICC for the first and second survey were 0.731 (CI 0.533 to 0.868; *p* < 0.01) and 0.942 (0.889 to 0.973; *p* < 0.01), respectively.

**Table 1.** Mean health utility ratings using a VAS (0 = ’Dead’, 100 = ’Full health’)

Health outcome descriptor	1^st^ Survey mean score (SD)	2^nd^ Survey mean score (SD)	Levene’s F statistic	*p*-value
Accessibility to Information	78 (18)	88 (9)	2.842	0.106
Awareness of Information	73 (17)	86 (14)	4.474*	0.045
Participation in Screening	79 (15)	84 (15)	0.458	0.505
Informed Decision Making	82 (16)	89 (11)	1.461	0.239
Satisfaction with Decision-Making	80 (12)	89 (12)	3.271	0.084
Confidence with Decision-Making	78 (18)	88 (14)	2.098	0.162
Abnormal Screening Result	62 (24)	78 (15)	4.519*	0.044
Recall for Assessment	64 (27)	74 (12)	1.387	0.208
False Positive Screening Result	68 (24)	69 (17)	0.032	0.861
Suspicious Indeterminate Calcification	64 (21)	68 (18)	0.250	0.622
False Positive Biopsy Result	67 (26)	56 (19)	1.387	0.252
Breast Cancer Detection	60 (31)	54 (19)	0.327	0.573
Breast Cancer Stage	60 (29)	52 (8)	0.783	0.386
Determination of Biomarker Status	68 (20)	66 (19)	0.069	0.795
Interval Breast Cancer	42 (28)	40 (15)	0.027	0.872
Over-Diagnosis & Over-Treatment	54 (23)	62 (18)	0.887	0.357
False Negative Screening Result	41 (29)	43 (18)	0.032	0.861
Radiation Exposure from Mammogram & Assessments Using Radiation	69 (26)	80 (19)	1.281	0.270
Provision of Surgical Therapy	62 (28)	54 (15)	0.743	0.395
Mastectomy	49 (26)	43 (16)	0.428	0.520
Provision of Medical Therapy	59 (28)	47 (11)	2.111	0.160
Provision of Radiotherapy	57 (26)	51 (13)	0.533	0.473
Provision of Chemotherapy	48 (25)	44 (9)	0.291	0.595
Other Cause Mortality	10 (20)	11 (22)	0.028	0.869

* p<0.05

## Discussion

### Key findings

This case study assessed the development of 24 health outcome descriptors in the context of the European guidelines for breast cancer screening and diagnosis. Thematic analysis of GDG interview feedback revealed that our novel and succinct format was useful and flexible for describing health outcomes. This finding builds upon prior research that identified short narratives as the preferred health outcome descriptor format by healthcare recipients [19].

Strengthening GDG understanding of outcomes and improving the transparency of guideline development methods were identified as the most impactful uses for health outcome descriptors. Changes made to the descriptors after the second round of GDG feedback may have resulted in a reduction in variance of the mean health utility scores rated with the VAS. This suggests that the process of health outcome descriptor development helped consolidate the values and preferences of the GDG, which is crucial for decision-making during the development of recommendations.

GDG members described the insufficient training on health outcome descriptor development methods and the time needed for this process as barriers to their participation. This study was carried out only because we established the need to explicitly describe outcomes that had already been considered by the GDG. However, by starting the study when the outcomes had already been prioritised for some questions by the GDG, we may have caused confusion among the GDG about the purpose of health outcome descriptors. Most GDG members had only been introduced to the GRADE approach in the context of the ECIBC guidelines, and so insufficient exposure to methods for outcome generation and importance rating as well as other core guideline methods in an ever-expanding field may have further contributed to the confusion regarding health outcome descriptors. In practice, we recommend that panel members receive training on guideline methodology, including health outcome descriptor development and their purpose. In addition, health outcome descriptors should be created before outcome importance is rated.

Online feedback was an effective and easy method for refining outcome-specific content for the developer group. The GDG’s serious concerns with the content of the first drafts suggest that a multi-disciplinary group of experts, involving representatives from the guideline panel, should be involved from the very beginning of health outcome descriptor development. For future efforts, we propose that a small multidisciplinary subset of the panel (no more than four people) be selected to work with a steering committee of guideline development methodologists to create and refine drafts of each health outcome descriptor. The steering committee should oversee population of the template by panel members to ensure that the structure is appropriate. The use of online or in-person feedback from panel members is appropriate to modify content. Ultimately, we believe that the steering committee should approve health outcome descriptors to be used for decision-making in the guideline.

Opinions on the appropriate balance of wording, reading level, and emotional sensitivity for end-users were varied. More research must be done on the specific needs of different end-user populations to conclude whether multiple tailored versions of health outcome descriptors are necessary or helpful. We propose that the steering committee declare intended end-user populations at the beginning of development, and use their professional judgement to ensure that wording, reading level, and emotional sensitivity is appropriate.

Participants also described having significant difficulty with the VAS for health utility rating because they felt that the health states anchoring the scale were inappropriate for rating some of the health outcome descriptors. This was particularly true of the outcomes ‘Accessibility to Information’, ‘Awareness to Information’, ‘Participation in Screening’, ‘Informed Decision Making’, ‘Satisfaction with Decision Making’, and ‘Confidence with Decision Making’. For these outcomes, the desired and undesired effects may have been perceived as independent from any physical health status.

Difficulties with the anchor health states are further supported by the health outcome descriptor for “Other-Cause Mortality” valued with a mean health utility score of 10. Given that the health outcome descriptor had similar content to the anchor health state “Dead” (which was visible during the rating exercise), it was expected to be valued at 0. The rating of 10 suggests that either there were some difficulties in completing the exercise, or it may have been due to a simple error. Relevant literature on the VAS describes it as being more acceptable and practical than other validated scaling methods [34]. Furthermore, the health states “dead” and “full health” are widely-used as anchors for scaling methods [35]. Given this, it is most likely that the difficulty with the survey was due to insufficient instructions, failure to understand instructions, or context bias resulting from rating the health utility of all health outcomes in the same survey. This was our reasoning for changing the instructions between surveys.

Although one participant provided feedback that the testing and treatment domain was inappropriate for outcomes related to preventive interventions, we did not make changes to the format. We believe that testing and treatment should be considered jointly and connected to healthcare interventions on a pathway that follows from a health state, even if no testing or treatment follows which in itself is important information.

### Limitations and strengths

A limitation of this study was that development of health outcome descriptors for most of the outcomes occurred after the GDG had already rated them for importance and included them in GRADE evidence tables. The development of the health outcome descriptors during the guideline development process may have caused confusion about the need and purpose of them, although the development need resulted precisely from disagreement arising about definitions of health outcomes.

Furthermore, health outcome descriptor development occurred in the context of only one breast cancer screening guideline, which limits our generalization to other panels and healthcare topics. Finally, for the utility rating this study had a small sample size which reduced the statistical power of our variance analysis.

A strength of this study is that all data was collected from a real-life guideline panel, which is rare among published literature on outcome descriptors. By conducting this case study in the context of a real guideline panel, our results can be used to inform outcome descriptor standardization efforts for guideline development, where we originally identified the problem of heterogeneity. We also carefully planned health outcome descriptor development methods and interaction with GDG members to capture reliable feedback at each stage of development. Collectively, our planning and analysis ensure that the results from this study can be used to inform all stages of health outcome descriptor development.

### Implications for practice

This study’s findings highlight the attitudes towards health outcome descriptor development and use among guideline panel members. Results suggest that guideline developers using health outcome descriptors should work with a multidisciplinary subgroup of panel members in a few rounds with online or in person feedback, to develop first drafts and final versions of the health outcome descriptors respectively. Prior to development, guideline panel members should be well informed, prepared, and trained on development methods and the GRADE approach accordingly. Our findings may help inform and guide future development of health outcome descriptors for guideline development. The ECIBC guideline health outcome descriptors will be used to better inform users of the outcomes that were considered in each of the healthcare questions by publishing them on the ECIBC website and they will also be used in decision support tools.

### Implications for research

Further research will show if multiple versions (e.g. policy maker, healthcare professional, etc.) of the health outcome descriptors for different target audiences are necessary, and how the reading level and wording of each version might be tailored to the different end-user populations. Our preference is that simple descriptors, that provide a common language for those providing health care and those receiving that care, should be used. A priori, there seems to be no logical reasons for a different language for different users. Using a common language will reduce the probability that misunderstandings, across different end-users, will occur.

For the use of health outcome descriptors to become more common in guideline development, there is a need to determine how guideline end-users make use of them, so instructions for their development can be altered accordingly. Most importantly, researchers should investigate whether health outcome descriptors do improve transparency and understanding of guideline methods for end-users, as some GDG members in this study suggested. Additional research efforts can build upon the present study by examining attitudes towards health outcome descriptor use by end-users, particularly healthcare recipients who may not have extensive medical knowledge [36]. Other research efforts might focus on how health outcome descriptors might be adapted for use for other purposes including, but not limited to, research and education.

Researchers should also concentrate efforts on determining the reliability of the VAS when rating the utility of health outcome descriptors, because we were unable to draw meaningful conclusions about this due to the limited statistical power in this study.

## Conclusion

This study describes the experiences of health outcome descriptor development for a health care guideline and provides guidance for future efforts in this area. Our standardized health outcome descriptor format may be useful for facilitating a common understanding of the outcomes chosen for the healthcare questions covered in a guideline, and thus improving the transparency of the guideline methods used. GDG members used health outcome descriptors with the VAS to improve precision of health utility ratings, but more research must be done to validate this method and reduce measurement error.

## Data Availability

The health outcome descriptors developed and/or analysed during the current study are available in an online repository (https://ms.gradepro.org/) upon searching the respective titles of the health outcomes used in this study. The datasets used and/or analysed during the current study are available from the corresponding author on reasonable request.

## Appendix

ECIBC guideline health outcome descriptors

1. **Accessibility to Information**
  **This health outcome descriptor refers to being able to access information about any breast cancer topic easily if you have been invited to participate in screening. It only considers the period for which you are receiving breast related healthcare.**
  **Accessibility to Information**

- **What you experience or feel:** You may need to invest effort to seek out information from different sources, including but not limited to your healthcare provider, personal contacts and the internet. You may feel satisfied if you obtained all the information you needed easily.
- **Time Horizon:** You may seek out information on breast cancer screening or on breast cancer a few weeks before you begin regular screening, or a few days after a test result has been communicated to you (or indeed at any other time). You may identify relevant information within minutes to hours depending on the accessibility of what you search for, and how you search for it.
- **Testing and Treatment:** The information which you access may affect your diagnostic and treatment experience in the context of shared decision making. Easy access to information may influence the type and frequency of diagnostic tests, but not screening tests, you may undergo. Depending on the quality of the information you obtain, your screening frequencies, and, if appropriate, diagnostic tests and treatment for your potential breast cancer may be positively or negatively influenced as well.
- **Consequences:** You may find screening and other clinical experiences enhanced by greater knowledge as a result of access to information. On the other hand, you may experience anxiety due to having only a partial understanding of screening, breast cancer, or the risk of suffering from it. Although accessible, the information you find may be inaccurate and in that case, you may make uninformed decisions.

2) **Awareness of Information**
  **This health outcome descriptor refers to being knowledgeable about any breast cancer topic during the period of time for which you are receiving any breast related healthcare for potential/confirmed breast cancer. You may receive information from your healthcare professional, health authorities, the internet, and other sources.**
  **Awareness of Information**

- **What you experience or feel:** If you are aware of information, you may feel satisfied with your breast healthcare.
- **Time Horizon:** You may start researching breast cancer and screening/diagnostic testing information a few weeks before your first screening/diagnostic test or immediately after a possible diagnosis of breast cancer or recall invitation. Your level of awareness about screening, breast cancer and diagnostic tests for breast cancer may increase over time.
- **Testing and Treatment:** Having a high level of awareness may impact the type and frequency of any diagnostic tests, but not screening tests, you may undergo. Depending on the quality of the information you obtain, your screening frequencies, and, if appropriate, diagnostic tests and treatment for your potential breast cancer may be positively or negatively influenced as well.
- **Consequences:** You may experience anxiety due to a partial understanding of screening, breast cancer, or the risk of suffering from it. Alternatively, you may feel more satisfied given that you are aware of the consequences of testing and treatment for early breast cancer.

3) **Participation in Screening**
  **This health outcome descriptor refers to participating in breast cancer screening or testing. In all situations, you will have an opportunity to express the value you place on the benefits and harms to health care professionals.**
  **Participation in Screening or Testing**

- **What you experience or feel:** You may receive a verbal or written invitation for mammography from a screening programme or a healthcare professional. The invitation will give you the details for having the mammography and information about the expected benefits and harms that you can obtain by participating in screening. Before or at the screening appointment, you can ask questions about this information and decide if you will participate in the screening programme. If you feel fully informed (described in a separate health outcome descriptor) you might feel satisfied with the decision-making process.
- **Time Horizon:** Once you decide to participate in a screening programme, it may take a few days, weeks, or months before you undergo the test. If you receive an invitation for screening, it will usually take some weeks.
- **Testing and Treatment:** Depending on the results of the tests, additional testing and, if breast cancer is diagnosed, subsequent treatment may be required, or you may not require additional testing until the next time you are invited or decide to participate. You may receive tests or treatments that you and your doctor have decided are appropriate for you.
- **Consequences:** If you undergo a recommended test and your decision is based on the information you received, you may be satisfied (what satisfaction may mean to you is addressed in a separate health outcome descriptor). If you are recalled for further assessment you may visit your healthcare professional again. If you are recalled for a further assessment, you will eventually be found to have or not have breast cancer. The clinical outcome may or may not extend your lifetime as a result of early detection of cancer.

4) **Informed Decision Making**
  **This health outcome descriptor refers to you and your healthcare professional, together making healthcare decisions based on as much relevant information as possible.**
  **Informed Decision**

- **What you experience or feel**: You might feel empowered, confident, and satisfied with the decision-making process and the decision itself.
- **Time Horizon:** You may become more informed on the subject of breast cancer, breast cancer screening, diagnosis and treatment during the period for which you are receiving breast cancer healthcare. The amount of external influence on your decisions may vary over time.
- **Testing and Treatment:** The amount of knowledge you have before making a decision may affect the type and frequency of testing and treatment you may undergo.
- **Consequences:** You may ignore or be unaware about breast cancer information outside your current knowledge. You make the decision that is right for you, based on all available evidence and bearing in mind your values, priorities and lifestyle. However, you and your loved ones may occasionally feel uncomfortable, because of differences between your personal understanding and the advice from your healthcare professional, or because the new information overturns opinions you held previously.

5) **Satisfaction with Decision Making**
  **This health outcome descriptor refers to the level of satisfaction you feel about the decision-making process and any decision that you and your healthcare provider have made about your breast cancer testing and/or treatment.**
  **Satisfaction with Decision Making**

- **What you experience or feel:** You may have the opportunity to provide input in your breast-related healthcare decisions. You may feel content with the process and the actual decision.
- **Time Horizon:** You may be content both immediately after information is presented to you and within a few days of making any decision related to testing and/or treatment. This feeling could disappear or change over time.
- **Testing and Treatment:** You may receive tests or treatments that are based on your informed decisions. Your satisfaction with the decisions made by you and your healthcare provider may affect the type and frequency of tests and/or treatments you undergo.
- **Consequences:** You may be satisfied with your breast healthcare. You may have less anxiety about your care and have a positive relationship with your healthcare provider.

6) **Confidence with Decision Making**
  **This health outcome descriptor refers to making a decision (with consultation from your doctor) about your breast cancer-related healthcare with high confidence.**
  **Confidence in Making Decisions**

- **What you experience or feel:** You may have the opportunity to provide input in your breast cancer-related healthcare decisions. With high confidence in your decisions, you may feel satisfied in the decision-making process. With little confidence, you may feel dissatisfied.
- **Time Horizon:** You may start making breast cancer testing decisions weeks before your first regular screening or diagnostic test. You may be confident from that point onward.
- **Testing and Treatment:** Your confidence in the decisions made by you (and your healthcare professional) may affect the type and frequency of any screening or diagnostic tests you may undergo.
- **Consequences:** Additionally, you may ignore or be unaware about breast cancer information outside your current knowledge. Despite being confident, your decision may be right or wrong for you. However, it is more likely to be right for you if you have confidence in your decision.

7) **Abnormal Screening Results**
  **This health outcome descriptor refers to any abnormal screening mammography result that requires you to be recalled for further diagnostic assessment. Your healthcare provider will organise this follow up (recall).**
  **Abnormal Screening Result**

- **What you experience or feel:** When you are informed (in person, by phone or by letter) that a suspicious abnormality has been identified on the screening mammogram you may be concerned and anxious.
- **Time Horizon:** You will receive the results of your test and/or be recalled for further assessment within 1–2 weeks of your screening mammogram being performed.
- **Testing and Treatment:** Further assessment may include additional imaging, and eventual biopsy, and/or other testing; all of which may be performed by a specialist healthcare professional in an assessment centre or hospital. If cancer is diagnosed, you will be referred for treatment based upon the stage of your breast cancer, tumour biomarker status, age, and your general health. You may also be treated for anxiety arising from the disease.
- **Consequences:** You and your loved ones may experience periods of stress and anxiety because of uncertainty associated with being recalled and going through the experience of additional assessment. Going to additional assessments may necessitate taking time off work or other inconvenience. If the results suggest the possible presence of breast cancer you will be advised to have additional testing, biopsy, and, if breast cancer is diagnosed, treatment. If you have a biopsy, this may have physical side effects (see health outcome descriptors 16, 18 and 19). You may feel relief if the assessment shows that the suspicious lesion turns out not to be cancer.

8) **Recall for Assessment**

**This health outcome descriptor refers to being recalled for further assessment due to abnormal mammographic findings (or technically inadequate mages) at the screening examination. Further assessment is needed to rule out or confirm breast cancer.**

**Recall for assessment**

- **What you experience or feel:** When you are informed (by phone and/or letter) that a suspicious abnormality has been identified on the screening mammogram you may be concerned and anxious.
- **Time Horizon**: You will receive the results of your test and/or be recalled for further assessment within 1–2 weeks of your screening mammogram being performed.
- **Testing and Treatment:** Further assessment may include additional imaging, and eventual biopsy, and/or other testing; all of which may be performed by a specialist healthcare professional in an assessment centre or hospital. If cancer is diagnosed, you will be referred for treatment based upon the stage of your breast cancer, tumour biomarker status, age, and your general health. You may also be treated for anxiety arising from the disease.
- **Consequences:** You and your loved ones may experience periods of stress and anxiety because of uncertainty associated with being recalled and going through the experience of additional assessment. Going to additional assessments may necessitate taking time off work or other inconvenience. If the results suggest the possible presence of breast cancer you will be advised to have additional testing, biopsy, and, if breast cancer is diagnosed, treatment. If you have a biopsy, this may have physical side effects (see health outcome descriptors 16, 18 and 19). You may feel relief if the assessment shows that the suspicious lesion turns out not to be cancer.

9) **False-Positive Screening Result**
  **This health outcome descriptor refers to the effects associated with having a screening mammogram that caused a recall for further assessment and therefore led you to believe you might have breast cancer when you do not.**
  **False-Positive Screening Result**

- **What you experience or feel:** When you are informed (by phone and/or letter) that a suspicious abnormality has been identified on the screening mammogram you may be concerned and anxious.
- **Time Horizon:** You will receive the results of your test and/or be recalled for further assessment within 1–2 weeks of your screening mammogram being performed.
- **Testing and Treatment:** Further assessment may include additional imaging, and eventual biopsy, and/or other testing; all of which may be performed by a specialist healthcare professional in an assessment centre or hospital. If you have a biopsy, this may have physical side effects (see health outcome descriptors 16, 18 and 19).
- **Consequences:** You and your loved ones may experience anxiety and resource use. When you receive the result that there is no breast cancer on assessment, you may feel relief.

10) **Suspicious Indeterminate Calcifications in Mammography**
  **This health outcome descriptor refers to the state of having a diagnostic mammography result that identifies calcifications, which might be suggestive of breast cancer.**
  **Suspicious Indeterminate Calcifications in Mammography**

- **What you experience or feel:** On your mammogram, a radiologist may detect calcifications suspicious of breast cancer. These radiological findings typically do not give symptoms. You may experience anxiety about the uncertainty of your diagnosis.
- **Time Horizon:** You will receive the results of your test and/or be recalled for further assessment within 1–2 weeks of your screening mammogram being performed.
- **Testing and Treatment:** Further assessment may include additional imaging, and eventual biopsy, and/or other testing; all of which may be performed by a specialist healthcare professional in an assessment centre or hospital. If you have a biopsy, this may have physical side effects (see health outcome descriptors 16, 18 and 19). Depending on whether breast cancer is diagnosed, you may be advised to have treatment for breast cancer.
- **Consequences**: You and your loved ones may experience anxiety after you have been recalled for further assessment and during the time until the diagnosis is concluded and the decision about whether or not to have treatment is agreed upon.

11) **False-Positive Biopsy Result**
  **This health outcome descriptors refers to the effects associated with having a biopsy result that led you to believe you might have breast cancer when you do not.**
  **False-Positive Biopsy Result**

- **What you experience or feel:** You think that you have breast cancer when in reality you do not. You may experience intense anxiety, and consequent physical symptoms such as sleeping problems, as a result of having to undergo a biopsy for a possible breast cancer. After you realize that you were given a false positive diagnosis you may experience relief and anger.
- **Time Horizon:** Times for identifying a false positive diagnosis vary according to the type of lesion and the procedures at your breast cancer assessment centre or hospital. A false positive diagnosis is likely to be identified within a few weeks of the biopsy. You may experience anxiety (among other symptoms) during the time you believe you have breast cancer. You may also continue to worry after being told that the result was inaccurate and that you do not have breast cancer.
- **Testing and Treatment:** The biopsy may take place in a breast assessment centre or hospital by a healthcare professional. Generally, false positive breast biopsies are very rare. As a result of the false positive biopsy, you may undergo surgery and removal of breast tissue. In very rare circumstances, your entire breast may be removed.
- **Consequences:** If you are having surgery, you may experience swelling, soreness of the skin or infection in the area of the tissue sample collection. You may experience unnecessary cosmetic damage to your breast and/or loss of your breast as a result of any surgery. You and your loved ones may experience anxiety and may feel frustrated due to unnecessary resource use.

12) **Breast Cancer Detection**
  **This health outcome descriptor refers to the correct diagnosis of breast cancer after a positive mammogram followed by further diagnostic assessment and tests.**
  **Breast Cancer Detection**

- **What you experience or feel:** When you are told you have breast cancer, you may experience considerable anxiety, which in turn may cause physical symptoms such as sleeping problems. However, you may feel relieved if your breast cancer was detected in an early stage. You may experience considerable uncertainty about whether your cancer is likely to develop and requires treatment.
- **Time Horizon:** The diagnosis of breast cancer is confirmed at the end of the assessment process. This includes full histopathological assessment of the tissue that has been removed from your breast. The whole process may take 1 to 4 weeks from obtaining the results of your screening mammogram. You may begin to experience emotional symptoms after receiving your screening result, indicating the possibility that you may have breast cancer.
- **Testing and Treatment:** After confirmation of breast cancer, your diagnosis and treatment options may be discussed by a multidisciplinary team. You may be referred for further diagnostic testing to determine the extent of the cancer in your body. The multidisciplinary team may propose a targeted treatment which may vary according to the stage of your breast cancer, tumour biomarker status, age and your general health.
- **Consequences:** During the time that your treatment plan is being formulated by the multidisciplinary team you may feel additional stress and anxiety.

13) **Breast Cancer Stage**
  **This health outcome descriptorrefers to the state of having any stage of breast cancer. An early stage indicates that the breast tumour is relatively small and has not spread to other parts of the body. This means that you may be offered less aggressive treatment and may have a better prognosis. A later stage indicates that the breast cancer has reached a greater size and/or has spread to regional lymph nodes or to other parts of the body. This usually requires more aggressive treatment and is associated with a worse prognosis. In addition to tumour size and extent, prognosis and treatment will also depend on the characteristics of the tumour including the histological grade and the biomarker status.**
  **Breast Cancer Stage**

- **What you experience or feel:** When you are told you have breast cancer, you may experience considerable anxiety, which in turn may cause physical symptoms such as sleeping problems. Due to presence of a breast cancer, you may also experience symptoms such as breast skin thickening, changes to breast size, shape or appearance or nipple discharge. If the cancer has spread to other parts of the body you may feel a lump under your arm or symptoms referable to body sites involved by tumour. These symptoms may not be present at all and if present may vary in intensity. If you have early stage breast cancer you may experience relief that it is been detected early.
- **Time Horizon:** The amount of time it takes for a cancer to go from an early to a late stage varies from months to years.
- **Testing and Treatment:** A sample of your breast tissue may be removed with a needle to make a diagnosis of your breast cancer (please see health outcome descriptors 16, 18 and 19). Further testing such as ultrasound, bone scan, computerised tomography, MRI and/or a PET scan (positron emission tomography) may be performed to assess the stage of your breast cancer. You will be referred for treatment based upon the results of the tests. Treatment will vary according to stage of your breast cancer, tumour biomarker status, age, and your general health.
- **Consequences:** Your breast cancer may shorten your life. Breast cancer detected at an early stage will be more likely to be cured than breast cancer detected at a late stage. You and your loved ones may experience anxiety.

14) **Determination of Tumour Biomarker Status in Biopsy**
  **The biomarker status of a tumour refers to the expression or otherwise of certain proteins by the the tumour. Expression of these features by a breast tumour predicts how the tumour may behave and more specifically how it might respond to specific treatment. The most important tumour biomarkers are expression of estrogen/progesterone hormone receptors and the HER2 (human epidermal growth factor receptor 2) oncogene. Some centres also assess the Ki67 index of the tumour to see how fast it is growing and to assist decision making regarding the need for chemotherapy.**
  **Determination of Tumour Biomarker Status**

- **What you experience or feel:** You do not feel the expression of a tumour biomarker. You may experience relief if your biomarker status suggests a relatively good prognosis or if the biomarker status allows a targeted therapy directed against the tumour. However, you might be concerned if the biomarker suggests a possibly worse outcome.
- **Time Horizon:** You will receive results of testing for the tumour biomarker within approximately 10 days of the biopsy procedure.
- **Testing and Treatment:** Your biomarker status will be determined using immunohistochemical and in situ hybridization techniques. The tests are performed in a histopathology laboratory. A multidisciplinary team will discuss your treatment options. The presence of certain biomarkers in a breast cancer will have an impact on the type of treatment that you will be offered. Expression of estrogen/progesterone receptors suggests you may benefit from endocrine therapy. Expression of HER2 suggests you may benefit from anti-HER2 therapy. If none of the biomarkers is expressed you may benefit from an alternative type of chemotherapy.
- **Consequences:** You may experience anxiety in the time between having a biopsy performed and receiving results of your biomarker status. The results will have an impact on the type of treatment you receive. They also influence your chances of being cured of breast cancer.

15) **Interval Breast Cancer**
  **This health outcome descriptor refers to having a diagnostic test correctly identify a cancer after you have had a screening test, with or without further assessment, which was negative for malignancy, either: before the next invitation to screening; or within a time period equal to the screening interval after you have reached the upper age limit for screening.**
  **Interval Cancer**

- **What you experience or feel:** When you are told you have breast cancer, you may experience considerable anxiety, which in turn may cause physical symptoms such as sleeping problems. You may feel relieved if your breast cancer was detected in an early stage. Due to the presence of breast cancer you may experience symptoms such as a breast lump, nipple discharge, skin thickening or a change in the size, shape or appearance of your breast. You may also feel concern that your tumour may have been present at the time of screening and was not detected.
- **Time Horizon:** This tumor may have become symptomatic in the period of time since your prior screening examination. The methods of assessment used to identify the tumor and confirm the diagnosis, including the time taken, are outlined in health outcome descriptors 16, 18, 19, 20, 21 and 22 above.
- **Testing and Treatment:** Following the mammogram, additional mammographic views, ultrasound, MRI and/or contrast enhanced mammography (CESM) may be performed for further assessment of your breast. This will be carried out in a hospital or in a breast centre. Treatment will vary according to the stage of your breast cancer, tumour biomarker status, age, and your general health.
- **Consequences:** Since the tumor was not visible at prior screening it might be fast growing and biologically more likely to spread. However, it is possible that your tumour is still at an early stage. Your breast cancer may shorten your life. Breast cancer detected at an early stage will be more likely to be cured than breast cancer detected at a late stage. You and your loved ones may experience anxiety.

16) **Over-diagnosis and Over-treatment**
  **In screening, it is possible to diagnose a breast cancer which is so slow-growing that it would never have been diagnosed in a person’s lifetime if the person had not been screened. The scientific term for breast cancer that would have not been diagnosed without screening is “over-diagnosis” of cancer. We cannot tell which cancers are of this type, however. Because it is unknown which cancers are over-diagnosed, treatment is the same as if it was not over-diagnosed. This is referred to as over-treatment. An over-diagnosed cancer is likely to be detected at an early stage.**
  **Over-diagnosis and over-treatment**

- **What you experience or feel:** When you are told you have breast cancer, you may experience considerable anxiety, which in turn may cause physical symptoms such as sleeping problems. However, you may feel relieved if your breast cancer was detected in an early stage. You may experience considerable uncertainty about whether your cancer is likely to develop and requires treatment.
- **Time Horizon:** The time between receiving the diagnosis due to a recall from screening and receiving treatment is the same whether or not the cancer is over-diagnosed. If treatment is confined to local therapy, it is completed in 6–8 weeks. Other therapy, such as hormone therapy can last several years. If you had not participated in screening, you would have remained unaware of the cancer and free of symptoms throughout your normal lifetime.
- **Testing and Treatment:** The screening mammography is performed in a breast screening centre by a healthcare professional. Due to suspicious findings on your mammogram, you will be called for further assessment at a breast cancer assessment centre or a hospital. Detection of the cancer will not be beneficial to your health because your tumour is of no clinical importance. You will be referred for treatment based upon the results of the assessment. Treatment will vary according to stage of your breast cancer, tumour biomarker status, age, and your general health.
- **Consequences:** Any treatment you receive may have side effects (described in other health outcome descriptors). You will have to return to your healthcare professional for additional diagnostic testing and treatment. You and your loved ones may experience anxiety and costs compared to if the breast cancer had never been diagnosed.

17) **False-Negative Screening Result**
  **This health outcome descriptor refers to receiving a negative screening result (no breast cancer) when you actually have a breast cancer. This is called a false negative screening result. Not all women become aware that they had a false negative screening result. This health outcome descriptor describes when they do become aware after subsequent diagnosis.**
  **False Negative Screening Result**

- **What you feel or experience:** When you find out that you did have breast cancer and it was missed, you are likely to feel anger, fear, and anxiety.
- **Time Horizon:** It may take months to years before you find out that you did have breast cancer when you were told you did not.
- **Testing and Treatment:** Following the discovery of your breast cancer later on, you may have to undergo treatment that is more intense than if the cancer had been detected right away, as the cancer may have developed to a more advanced stage.
- **Consequences:** The consequences of late detection of a slow growing breast cancer will probably be not substantial with respect to treatment and prognosis. However, if the breast cancer has grown, your predicted outcome is likely worse than if it had been diagnosed at the screen. Survival from breast cancer that has a false-negative diagnosis may be worse compared to women with screen-detected breast cancer, but comparable to women who do not attend screening.

18) **Radiation Exposure from Mammograms & Other Assessments Using Radiation**
  **This health outcome descriptor refers to being exposed to any dose of radiation from undergoing a mammographic examination and any other related assessments only. It does not refer to therapeutic radiation.**
  **Radiation Exposure from Mammograms & Other Assessments Using Radiation**

- **What you experience or feel**: You do not feel the radiation itself. However, you may be anxious if you are not aware that the radiation dose is low or if you feel concerned at the prospect of any radiation dose associated with the examination.
- **Time Horizon:** Considering the low doses of radiation, no short-acting effects occur. In extremely rare cases, exposure to radiation may induce cancer in your breast. This may take many years.
- **Testing and Treatment:** You will be brought to a mammography device so images of your breast can be taken. Your breast will be placed on a plate and compressed to have a mammogram. Compression is needed to flatten the breast which will keep the radiation dose as low as is reasonably achievable.
- **Consequences:** Exposing your breast to radiation may induce cancer in the breast tissue. The scale of the harm is extremely small and difficult to quantify. It will increase with the number of mammograms over a lifetime.

19) **Provision of Surgical Therapy**
  **This health outcome descriptor refers to the state of undergoing surgery to the breast or axilla. This includes breast conserving surgery (removal of a breast lump with a rim of surrounding tissue), mastectomy (complete removal of your breast), open biopsy (removal of a small piece of tissue from your breast for diagnosis) and axillary surgery (removal of one or more lymph nodes, including the sentinel lymph node). It does not refer to any combination therapy.**
  **Provision of Surgical Therapy**

- **What you experience or feel:** You may experience anxiety and fear because of the procedure that will be performed. If breast conserving surgery (lumpectomy or quadrantectomy) or mastectomy is performed, you may experience loss of part or all of your breast and that may have an influence on your physical and psychological well-being. Preparation for surgery may involve other examinations and tests.
- **Time Horizon:** Surgery will be planned and scheduled. It may take weeks (or months if you receive chemotherapy prior to surgery) before the surgery is performed. The time taken for the operation will vary depending on the type of surgery and will be longer if you undergo reconstructive surgery at the same time.
- **Testing and Treatment:** All surgeries will be performed in an operating room. For breast conserving surgery or a mastectomy, you will be given general anesthesia, so you will be asleep. During the surgery, 1–2 incisions may be made in your breast. Some of your breast tissue (or entire breast) and, lymph nodes, and/or chest muscle may be removed depending on the type and stage of your cancer. This will be discussed with you by your surgeon before surgery. Following surgery, a histopathologist will examine the breast and axillary tissue that has been removed to analyze the tumour with regard to size, grade, type etc. The histopathologist will also examine the lymph nodes to see if the tumour has spread to these.
- **Consequences:** After the procedure, you may experience bruising, infection, haematoma, and/or tenderness of the breast. In rare cases, you may experience collapse of the lung. Additionally, you may have discomfort, inconvenience, embarrassment, and reduced self-esteem because of the loss of all or part of your breast, although this may be mitigated by reconstructive surgery.

20) **Mastectomy**
  **This health outcome descriptor refers to having any type of mastectomy performed. This is usually accompanied by removal of one or more axillary lymph nodes.**
  **Mastectomy**

- **What you experience or feel:** Before surgery you may be anxious and afraid. After surgery, you may experience pain. You may be concerned about the loss of your breast and how it will appear to other people.
- **Time Horizon:** The procedure takes approximately 2–3 h. It may take longer if reconstruction of your breast is included as part of the surgical procedure. You will be admitted to a hospital and stay for approximately 1–3 days if there are no complications. The remainder of your recovery may take place in your home. Your discomfort will disappear over the next weeks.
- **Testing and Treatment:** Your mastectomy will be performed by a breast surgeon or senologist at a hospital. You will be put under general anesthesia, so you will be asleep. During the surgery, a cut will be made into your breast and armpit (axilla), according to your pre-surgical discussion with your breast surgeon or senologist. Axillary lymph nodes will likely be removed in addition to your breast.
- **Consequences:** The planning of the procedure may make you feel anxious. After the procedure, you may experience pain related to the wound, bruising and breast tenderness. Occasionally you may experience infection, haematoma, and rarely lung collapse. You will not be able to conduct physical exercise or heavy lifting for a few weeks after the surgery. Additionally, you may have long-term discomfort, inconvenience, embarrassment, expenses, and reduced self-esteem for cosmetic reasons, although this may be mitigated by reconstructive surgery.

21) **Provision of Medical Therapy**
  **This health outcome descriptor refers to the state of receiving medical therapy for breast cancer treatment. This includes, but is not limited to chemotherapy or hormonal therapy. Counselling and psychological evaluation may be provided to support the psychological burden of breast cancer.**
  **Provision of Medical Therapy**

- **What you experience or feel:** During the course of the treatment you may experience anxiety, fear, or a feeling or sense of confusion.
- **Time Horizon:** You may begin treatment as early as within one week of diagnosis. The duration of your treatment will vary according to the type of treatment you are receiving.
- **Testing and Treatment:** Medical treatments may include pills, injections and infusions. More invasive or aggressive treatments will take place in your breast cancer centre or hospital. You may be referred to a psychiatrist for evaluation or psychotherapy in combination with your medical therapy.
- **Consequences:** During the course of treatment, you may have to visit your healthcare professional frequently. Medications and various forms of treatment may cause side effects (described in other health outcome descriptors).

22) **Provision of Radiotherapy**
  **This health outcome descriptor refers to the state of receiving radiotherapy after surgery to reduce the risk of local breast cancer recurrence. This includes, but is not limited to external beam breast radiation, internal breast radiation, or brachytherapy. It does not refer to any combination therapy.**
  **Provision of Radiotherapy**

- **What you experience or feel:** You may experience feelings of anxiety when you undergo radiotherapy. Additionally, you may experience fatigue, or skin irritation at the site of radiotherapy.
- **Time Horizon:** You may experience symptoms within hours of exposure. However, generally the amount of time between radiation and the onset of radiation exposure symptoms is dependent upon how much radiation you have been exposed to. Symptoms may occur months or even years after the treatment.
- **Testing and Treatment:** You will visit a radiotherapy clinic for your radiotherapy. During each session of treatment, you will lie under a machine that applies radiation to your breast to kill cancerous cells, potentially still present after surgery.
- **Consequences:** From hours to years after receiving radiotherapy at your breast, you may experience infections, itchiness, bone weakening, skin cancer, and low blood pressure after radiation exposure. Additionally, very few women may develop lung symptoms such as breathlessness, cardiovascular disease as a result of cumulative radiation exposure to the left breast or have a small risk of other cancers.

23) **Provision of Chemotherapy**
  **This health outcome descriptor refers to the state of receiving chemotherapy alone.**
  **Provision of Chemotherapy**

- **What you experience or feel:** During the course of the treatment you may experience fatigue, pain, hair loss, mouth and throat sores, diarrhea, nausea, vomiting, constipation, bleeding, infections and nervous system effects such as numbness or tingling. The severity of your symptoms may vary from very little to severe.
- **Time Horizon:** Each individual chemotherapy treatment may last up to 3 or 4 h. You may experience nausea and vomiting within a few hours of every chemotherapy treatment. Other symptoms may occur within days to months.
- **Testing and Treatment**: For oral chemotherapy, you can take the medication yourself at home. If you are receiving intravenous therapy you will be given the drug through a needle inserted into one of your veins. This type of chemotherapy is normally performed in your healthcare professional’s clinic. You will have physical examinations and blood samples taken. You may also have further radiological tests to assess response to treatment. If you suffer a complication, e.g. an infection, you will receive treatment for it.
- **Consequences:** During the course of treatment, you may have to visit your healthcare professional frequently and your quality of life may decrease. You may experience anxiety. Rarely you may suffer permanent impairment from a complication of treatment.

24) **Other-Cause Mortality**
  **This health outcome descriptor refers to the state of being dead due to factors unrelated to your breast cancer. It does not refer to the process of dying or outcomes that precede it (e.g. the breathlessness related to it or pain).**
  **Other Cause Mortality**

- **What you experience or feel:** You are dead and feel no pain. You may experience symptoms prior to dying from causes other than breast cancer but you do not feel those when you are dead.
- **Time Horizon:** Before you die, you experience other states of disease of varying duration.
- **Testing and Treatment:** Tests and treatment will have ceased.
- **Consequences:** You lose your vital bodily and mental functions, ending your life.

*Note: all outcomes were rated as critical or important. All descriptors presented here can be used for rating the importance of the outcome or the utility. For practical purposes, guideline developers may begin with developing a briefer version of a health outcome descriptors to rate the importance and expand when an outcome is deemed important or critical. In that latter scenario concise and more detailed versions of the health outcome descriptors will exist.*

## Abbreviations

GRADE: Grading of Recommendations, Assessment, Development and Evaluations
ECIBC: European Commission Initiative on Breast Cancer
GDG: Guidelines Development Group
VAS: Visual Analogue Scale
JRC: Joint Research Centre
